# Dementia assessment and management in primary care settings: a survey of current provider practices in the United States

**DOI:** 10.1186/s12913-019-4603-2

**Published:** 2019-11-29

**Authors:** Alissa Bernstein, Kirsten M. Rogers, Katherine L. Possin, Natasha Z.R. Steele, Christine S. Ritchie, Joel H. Kramer, Michael Geschwind, Joseph J. Higgins, Jay Wohlgemuth, Rick Pesano, Bruce L. Miller, Katherine P. Rankin

**Affiliations:** 1 Philip R. Lee Institute for Health Policy Studies, University of California, San Francisco, San Francisco, USA; 2 Global Brain Health Institute, University of California, San Francisco, San Francisco, CA USA; 3 Department of Neurology, University of California San Francisco, San Francisco, USA; 4 University of Washington School of Medicine, Seattle, WA USA; 5 Division of Palliative Care and Geriatric Medicine, Department of Medicine, Massachusetts General Hospital, Boston, MA USA; 6 Quest Diagnostics, Secaucus, NJ USA

**Keywords:** Primary care, Dementia, Neurocognitive disorders, Diagnosis, Care management

## Abstract

**Background:**

Primary care providers (PCPs) are typically the first to screen and evaluate patients for neurocognitive disorders (NCDs), including mild cognitive impairment and dementia. However, data on PCP attitudes and evaluation and management practices are sparse. Our objective was to quantify perspectives and behaviors of PCPs and neurologists with respect to NCD evaluation and management.

**Methods:**

A cross-sectional survey with 150 PCPs and 50 neurologists in the United States who evaluated more than 10 patients over age 55 per month. The 51-item survey assessed clinical practice characteristics, and confidence, perceived barriers, and typical practices when diagnosing and managing patients with NCDs.

**Results:**

PCPs and neurologists reported similar confidence and approaches to general medical care and laboratory testing. Though over half of PCPs performed cognitive screening or referred patients for cognitive testing in over 50% of their patients, only 20% reported high confidence in interpreting results of cognitive tests. PCPs were more likely to order CT scans than MRIs, and only 14% of PCPs reported high confidence interpreting brain imaging findings, compared to 70% of specialists. Only 21% of PCPs were highly confident that they correctly recognized when a patient had an NCD, and only 13% were highly confident in making a specific NCD diagnosis (compared to 72 and 44% for neurologists, both *p* < 0.001). A quarter of all providers identified lack of familiarity with diagnostic criteria for NCD syndromes as a barrier to clinical practice.

**Conclusions:**

This study demonstrates how PCPs approach diagnosis and management of patients with NCDs, and identified areas for improvement in regards to cognitive testing and neuroimaging. This study also identified all providers’ lack of familiarity with published diagnostic criteria for NCD syndromes. These findings may inform the development of new policies and interventions to help providers improve the efficacy of their decision processes and deliver better quality care to patients with NCDs.

## Background

In the United States, primary care providers (PCPs) are typically the first to screen and evaluate patients with neurocognitive disorders (NCDs) such as mild cognitive impairment and dementia. Currently, in the primary care setting, as many as two-thirds of people with dementia may be misdiagnosed (i.e., the NCD syndrome and neurologic etiology are misidentified), and there is often a significant delay between symptom onset and diagnosis [1–5]. Accurate diagnostic classification of an NCD determines prognosis and symptomatic treatment, and is necessary to identify the 2% of NCD cases with a reversible underlying condition [2, 6]. Accurate and early diagnosis can also enable patients and families to make important medical, legal, and financial decisions for the future, develop safety measures in the home, participate in clinical trials, and set goals for how to live with the disease [7–10]. Additionally, accurate diagnosis can help providers and patients work together to make lifestyle modifications, such as diet, exercise, and medications, in order to best manage symptoms [11–13].

PCPs have identified systems- and health service-level barriers to the assessment, diagnosis, and management of their patients with NCDs. These barriers include lack of technological, financial, and human resources, as well as inadequate time and expertise to educate patients and families after a diagnosis of dementia [4, 14, 15]. PCPs report difficulty addressing NCD symptoms in the context of common comorbidities such as cardiovascular disease [6], and both PCPs and patients commonly report trouble accessing specialists [15]. In fact, more than half of patients referred to an NCD specialist following a positive screen in the primary care setting do not obtain further evaluation [16, 17]. PCPs, not specialists, carry the principal responsibility of diagnosing and caring for the majority of patients with NCDs, yet experience major barriers to managing patients [17, 18] Furthermore, there is disagreement about what constitutes an appropriate evaluation and which screening tools best fit a patient’s characteristics and presenting symptoms. While some efforts have been made to create consensus protocols in this area, particularly for specific syndromes such as Alzheimer’s disease (AD), no comprehensive guidelines for NCD evaluation or choice of tools have been widely adopted [19, 20]. For example, while there is broad support among neurologists for using MRI rather than CT for brain imaging, the field has never formalized this standard of care^21, 22.^

In this study, we surveyed a national sample of PCPs in order to characterize their experiences and practices with respect to NCD evaluation and management. To better identify how PCP behaviors and attitudes differ from those of specialists trained in NCD diagnosis and care, a sample of neurologists was also surveyed. Given the systems-level barriers in primary care and the uncertainty around standards of care, our goal in this paper is to provide insights into provider attitudes and behaviors as a way to understand where guidelines may be most useful, and to identify specific areas to focus training and policy interventions. A clearer understanding of PCP attitudes, practices, and perceived barriers may guide efforts to better support their ability to provide quality care for the growing population of patients with NCDs and to improve health services for people with suspected NCDs.

## Methods

### Participants

Eligible participants were contacted via email invitation from a proprietary database of 5 million panelists. PCPs and neurology specialists who accepted the invitation were provided a link to a web page describing the project**,** anonymity policy, compensation appropriate based on specialty (between $23–$42 per survey), and participation requirements. Those who agreed to participate provided written consent in a form provided through the survey link and answered eligibility screening questions confirming that they 1) were a neurologist or PCP, and 2) saw more than 10 patients per month over the age of 55. The first 100 eligible PCP and the first 50 neurologist respondents were enrolled and completed the survey. The study was approved by the Institutional Review Board at the University of California, San Francisco (UCSF).

### Survey

The survey was designed by academic clinical researchers at UCSF specializing in dementia diagnosis and care, and was constructed to reflect domains from a behavior change model for implementation research called the Behavior Change Wheel Framework (BCW) [23]. The BCW is a theory-driven approach that uses a synthesis of 19 behavior change models. The BCW framework enabled us to understand provider behaviors, which can ultimately lead to the development of strategies for improvement and intervention targets that will most likely bring about clinic- and provider-level change [24]. Specifically, the survey measured providers' confidence, satisfaction, attitudes, and behaviors towards performing activities related to the diagnosis and management of NCDs, as well as their clinical practice characteristics (see Additional file 1). Providers responded to questions about the frequency with which they engage in specific activities related to the assessment, diagnosis, and care of their patients with NCDs, the usefulness of specific tools and tests (cognitive assessments, labs, imaging, screens), and what tools and knowledge would improve their ability to assess, correctly diagnose, and provide high-quality ongoing care for all common NCDs. Information on practice setting and typical patient demographics was also collected.

### Statistical analysis

Providers’ demographic data and outcome measures were summarized with descriptive statistics. Mean differences between PCPs and neurologists on stratified confidence rankings were tested using Wilcoxon Rank Sum Tests, accepting significance at *p* < 0.05 (IBM Corporation, SPSS Version 24, Armonk, NY).

## Results

### Provider and patient characteristics

#### Provider and practice characteristics

Table 1 shows the stratification of PCPs and neurologists according to their practice setting. The majority of PCPs surveyed worked in private group practices, private individual practices, or academic medical centers. Neurologists predominantly worked in private group practices or in academic medical centers.

**Table 1:** Characteristics of the respondents surveyed (*N* = 150)

Survey Question	PCPs (*n* = 100)		Neurologists (*n* = 50)	
	*Number*	*%*	*Number*	*%*
Practice Setting^a,b^				
Academic	15	13.8	20	36.4
Accountable Care Organization	5	4.6	1	1.8
Community Health Center	9	8.3	5	9.1
Federally qualified health care center	6	5.5	1	1.8
HMO	4	3.7	1	1.8
Private Individual Practice	19	17.4	4	7.3
Private Group Practice	45	41.3	23	41.8
Other	6	5.5	0	0
Years in practice post–residency, mean (SD)	18.9 (10.4)	–	14.7 (9.0)	–
Providers who use electronic medical records	79	79	43	86

^a^ The denominator for percentage calculations is 109, reflecting multiple practice settings for 6 clinicians

^b^ The denominator for percentage calculations is 55, reflecting multiple practice settings for 2 clinicians

#### Typical patient characteristics

Table 2 shows that both PCPs and neurologists treated an average of about 150 patients over age 55 per month, about a quarter of whom were insured by Medicaid. Neurologists diagnosed mild cognitive impairment (19.5 new cases per month, SD = 18.6) and dementia (17.7 new cases per month, SD = 18.2) significantly more often than did PCPs (11.7 ± 17.4 and 9.8 ± 15.4, respectively). Neurologists reported managing care for an average of 85 NCD patients per month, with 57% of their patients over age 55; in contrast, PCPs managed an average of 51 NCD patients per month, with about one third of their patients over age 55.

**Table 2:** Providers’ reported patient characteristics

Survey Question	PCPs (n = 100)		Neurologists (n = 50)		
	Mean (SD)	Median	Mean (SD)	Median	*p* value^a^
Patients treated per month, number	295.3 (179.1)	300	283.8 (157.6)	250	0.701
Patients treated per month > 55 years old, number	156.4 (113.8)	127.5	150.1 (105.7)	127.5	0.742
Patients insured by Medicaid, %	23.2 (22.2)	15.0	25.9 (23.0)	20.0	0.488
New cases of mild cognitive impairment diagnosed/month	11.7 (17.4)	5	19.5 (18.6)	14.5	0.012
New cases of dementia diagnosed/month	9.8 (15.4)	2.5	17.7 (18.2)	10	0.005
Number of patients currently managed for mild cognitive impairment	27.6 (27.8)	20	41.4 (30.5)	34.5	0.006
Number of patients currently managed for dementia	23.1 (26.4)	10	43.4 (30.8)	40	< 0.001

NS: Not significant

^a^ type III sum of squares

### Provider confidence

#### Confidence in providing general medical care

PCPs and neurologists reported similar levels of confidence (no significant difference) in managing the general medical care of NCD patients, with between 46 and 64% of providers reporting high confidence levels.

#### Confidence recognizing and diagnosing NCDs

PCPs reported significantly lower confidence levels than neurologists in both the ability to correctly recognize that an NCD is present, and the ability to distinguish among specific NCD syndromes (progressive supranuclear palsy, behavioral variant frontotemporal dementia, etc.) (*p* < 0.001). Only 21% of PCPs reported high confidence levels in their ability to correctly recognize when a patient had an NCD (mean 4.3, SD 1.3 on a Likert scale of 7, with 1 as low and 7 as high), and 13% reported high confidence levels in providing a specific NCD diagnosis (mean 3.4, SD 1.6), compared to 72% (mean 5.5, SD 1.4) and 44% (mean 5.0, SD 1.4) respectively for neurologists.

### Typical PCP practices and perceived barriers

#### Referrals

Table 3 shows that PCPs referred a large percentage of patients with suspected NCDs to specialists. Fifty-four percent of PCPs referred more than half of their patients with suspected NCDs to a neurologist or other specialist for a dementia evaluation, with no PCPs reporting that they elected to do the workup in-house with 100% of patients. Sixty-six percent of PCPs referred more than half of their patients with suspected NCDs for neuropsychological testing with a specialist. Only 1% of PCPs reporting never referring patients to neuropsychological testing.

**Table 3:** Frequency of Provider Practices

Measure	PCPs (*n* = 100)	Neurologists (*n* = 50)
	%	%
Frequency in which provider or staff refers patients to a neurologist or other specialist for a dementia workup		
Greater than 50% of patients with cognitive concerns	54	22
Less than or equal to 50% of patients with cognitive concerns	46	52
Never	0	26
Frequency in which provider or staff refers a patient with suspected neurocognitive disorders for neuropsychological testing with a specialist		
Greater than 50% of patients with cognitive concerns	66	44
Less than or equal to 50% of patients with cognitive concerns	33	52
Never	1	4
Frequency with which provider or staff evaluates by other clinical methods (history and examination) but without standardized tools		
Greater than 50% of patients with cognitive concerns	43	42
Less than or equal to 50% of patients with cognitive concerns	54	48
Never	3	10
Frequency in which laboratory panels for reversible causes of cognitive impairment (B12, TSH, etc.) are included if no prior lab work has been done		
Greater than 50% of patients with cognitive concerns	84	86
Less than or equal to 50% of patients with cognitive concerns	12	14
Uses other providers’ data	3	0
Never	1	0
Frequency in which standardized depression screen is administered to patients		
Greater than 50% of patients with cognitive concerns	62	48
Less than or equal to 50% of patients with cognitive concerns	33	42
Never	4	8
Frequency in which provider or staff administers a standardized cognitive screening test to patients with cognitive concerns		
Greater than 50% of patients with cognitive concerns	50	66
Less than or equal to 50% of patients with cognitive concerns	41	28
Never	5	4
Who most often administers cognitive screening tests to patients?		
PCP	53	60
RN or NP	15	10
PA or other medical staff member	17	12
Neuropsychologist	13	18
Other	2	0

#### Assessment and diagnostic testing

Forty-three percent of PCPs reported that with more than half of their patients with suspected neurocognitive impairment, they do not utilize any standardized diagnostic tools such as cognitive testing or imaging, and rather rely on evaluating solely by history and examination (compared to 42% of neurologists) (Table 3).

#### Laboratory testing for reversible causes of dementia

Eighty-four percent of PCPs indicated that they order lab panels for common reversible causes of cognitive impairment such as B12 and thyroid stimulating hormone in more than half of patients suspected to have an NCD (compared to 86% of neurologists).

#### Depression screens

PCPs were more likely than neurologists to administer depression screens. Sixty-two percent of PCPs reported administering a standardized depression screen to over half of their patients with cognitive concerns, compared to 48% of neurologists.

#### Cognitive assessments

Fifty-percent of PCPs reported that they, or their staff, administered a standardized cognitive screening test (e.g., the Mini-mental State Exam [MMSE] or the Montreal Cognitive Assessment [MOCA]) to over half of their patients with cognitive concerns, compared to 66% of neurologists. Fifty-three percent of PCPs reported administering the test themselves, while 15% gave the task to a nurse, and 17% assigned the task to a physician’s assistant or other medical staff member. Thirty-five percent of PCPs found standardized cognitive screens highly useful for identifying whether a patient had an NCD; only 4% reported that these screens would not be of value to them.

Twenty percent of PCPs were highly confident in interpreting cognitive testing results, while 13% had low confidence. In comparison, 68% of neurologists were highly confident in interpreting cognitive testing results, while none reported low confidence.

When asked to identify reasons they might choose not to conduct or order cognitive testing, 45% of PCPs believed cognitive impairment could be adequately assessed through the clinical interview and observation, without the need for standardized cognitive assessment tools. Other barriers to performing cognitive testing were identified much less frequently by PCPs, including lack of access to neuropsychological testing (6.8%), financial expense (12.7%), and lack of time (5.9%).

#### Imaging

**Fig. 1:**
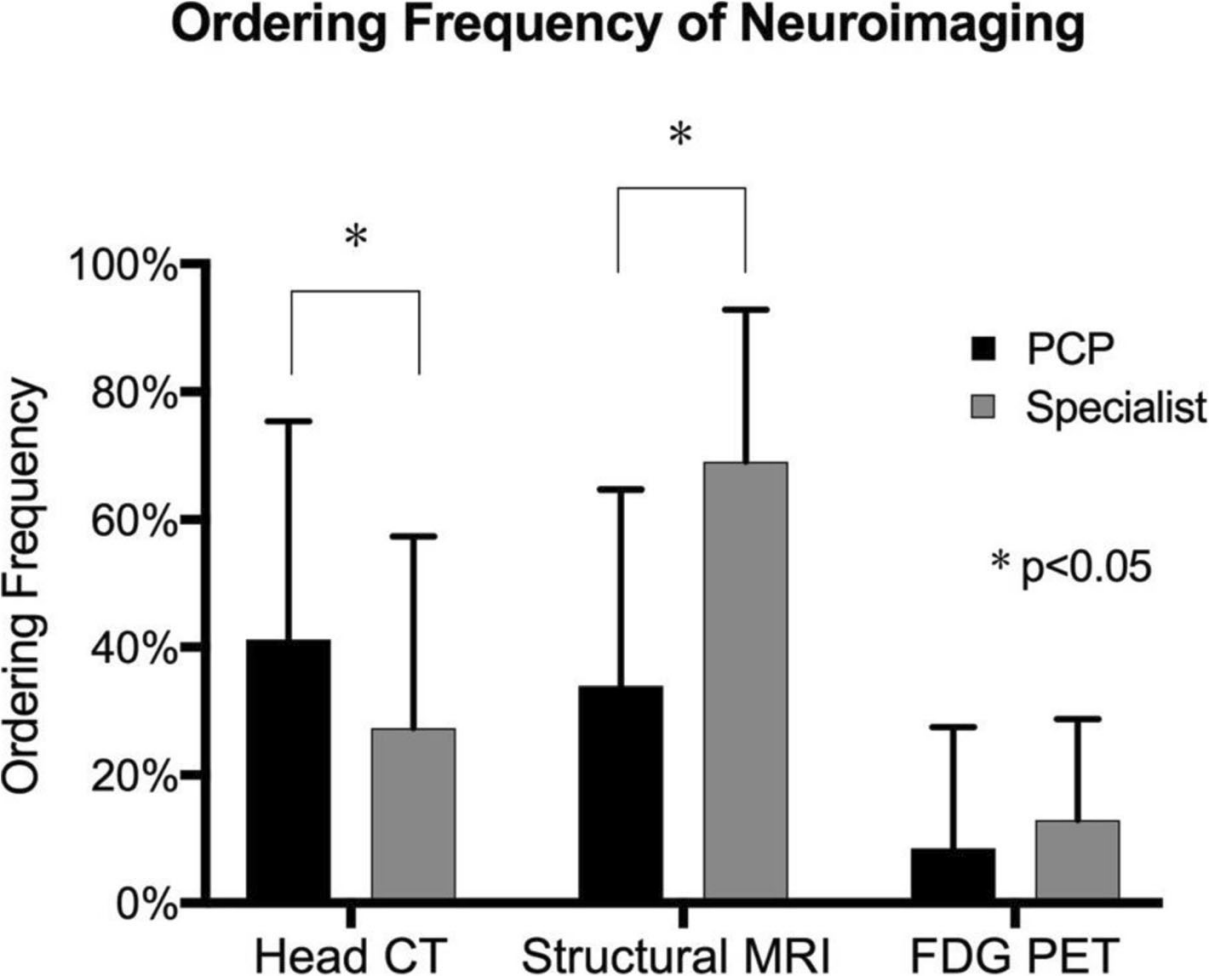
Frequency of ordering particular neuroimaging studies during dementia evaluation for patients with no prior imaging results

Some of the largest practice discrepancies between PCPs and neurologists occurred in the domain of brain imaging (Fig. 1). Only 15% of PCPs found brain scans to be highly useful in making a dementia diagnosis, as compared to 40% of neurologists. Furthermore, only 14% of PCPs reported high confidence in interpreting brain imaging findings, compared to 70% of neurologists. While neurologists most frequently ordered structural MRI (69%), only 34% of PCPs reported ordering structural MRI (*p* < 0.05) and instead favored brain CT (41%, compared to 27% of neurologists; *p* < 0.05). Both PCPs (9%) and neurologists (13%) rarely ordered FDG PET imaging.

When asked to identify particular barriers to use of brain imaging, 24% of PCPs identified a lack of familiarity interpreting MRI results as a barrier, compared to 3% of neurologists. Additional barriers included the expense of neuroimaging (19% of PCPs), the time it takes to obtain neuroimaging (7% of PCPs), neuroimaging results not changing diagnosis (15% of PCPs), and the value of neuroimaging based on their interpretation of practice guidelines (11% of PCPs).

### Beliefs about practice improvement

**Fig. 2:**
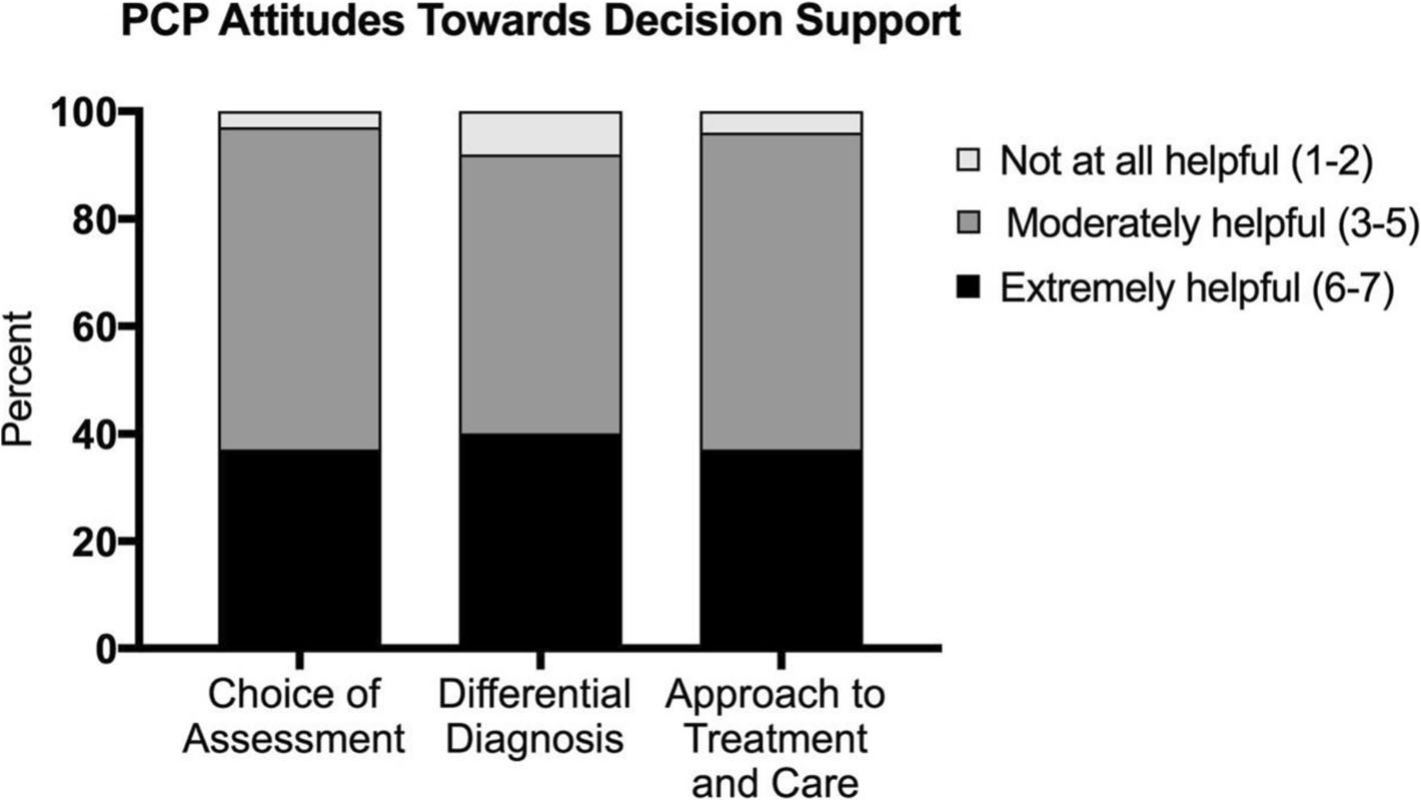
PCP mean Likert scores rating the helpfulness of decision support tools in choosing assessment procedures, how to perform differential diagnosis of neurocognitive disorder syndromes, and how to approach treatment and care of patients with different neurocognitive disorders

Thirty-one percent of PCPs reported that they would be highly likely to treat more patients with NCDs rather than referring them to specialists if decision support tools were available in their practices (Fig. 2). Thirty-seven percent reported they were highly likely to find decision support helpful for guiding their choice of assessment tests for NCDs. Forty percent of PCPs reported they were highly likely to find decision support helpful for guiding the diagnostic decision process, and 37% reported they were highly likely to find decision support helpful for guiding their patient management and ongoing care.

## Discussion

In this study we characterized current primary care provider and neurologist practices in the United States with respect to the assessment, diagnosis, and management of patients with NCDs, and identified areas to target for practice and policy improvement. There is a great deal of uncertainty and disagreement around guidelines for NCD evaluation and the best tools to use. Our goal in this paper was to identify specific areas, based on provider attitudes, behaviors, and practices, where it might be best to focus attention when creating standardized guidelines, training, and policy interventions for both primary care providers and for neurologists.

Our most notable findings were the lack of confidence and reported ability of PCPs to effectively implement and interpret cognitive testing and neuroimaging for the detection and diagnosis of NCDs. PCPs did report similar levels of frequency to that of specialists in ordering laboratory testing and in their confidence managing the general medical care of patients with NCDs. PCPs were even more likely to order depression screens, an important part of the NCD evaluation [25, 26]. However, their confidence and clinical practices diverged from specialists when performing other medical procedures that can provide early and accurate diagnosis of NCDs. Fifty-four percent of PCPs referred more than half of their patients with suspected NCDs to specialists (neurologists or neuropsychologists), rather than testing and diagnosing themselves, despite the fact that half of those patients are unlikely to actually follow through with that referral and obtain additional care [16, 17].

PCPs are often the sole care providers for individuals with NCDs, yet, as noted in other studies, frequently take a reactive rather than proactive approach to dementia diagnosis and care, and may underutilize available evaluation and management tools [15]. Prior investigations have found that primary care providers report issues of time, difficulty accessing and communicating with specialists, low reimbursement, difficulty connecting with service agencies, and lack of interdisciplinary teams as major barriers to the diagnosis and management of dementia in their practice settings [14, 15, 17, 27]. In our study PCPs also reported issues with time, reimbursement, and making referrals to specialists. However, our more comprehensive survey identified additional challenges for primary care providers in regards to the NCD evaluation process, including the use of cognitive testing, brain imaging, and standardized diagnostic criteria. Our findings suggest that the evaluation process is a domain where substantial guideline, training, and policy improvements can be made.

Surprisingly, we also found fewer practice differences between PCPs and neurologists in the areas of cognitive testing, imaging, and familiarity with diagnostic criteria than might be expected. For example, in the initial evaluation of patients with cognitive concerns, 43% of PCPs and 42% of neurologists reported that with more than half of their patients with suspected neurocognitive impairment they do not implement formal cognitive testing with standard diagnostic tools. This finding raises concerns for those who believe that a specialty evaluation for a neurocognitive disorder should include cognitive testing.

### Bridging gaps in care: training and policy implications

While there is still considerable uncertainty around standards for syndrome-agnostic NCD evaluation, multiple consensus guidelines recommend cognitive testing as an integral part of all NCD evaluations [19, 20, 28–30]. Yet, we found that 50% of PCPs used cognitive screens or tests in only half of their patients with suspected NCDs, and only 20% reported they had high confidence in their ability to interpret cognitive testing results compared to 68% of specialists. The most common cognitive screening tools currently used in primary care environments for the detection of cognitive symptoms are the MMSE [31] and the Mini-Cog [14]. However, novel and validated cognitive assessment tools are rapidly proliferating, but are often unknown to PCPs [10]. These new tools can evaluate multiple distinct cognitive domains (e.g., distinguishing memory, executive, language, and visuospatial problems) and are administered in the same amount of time as older screens (i.e., 10 min or less [10, 32–36]). PCPs may benefit from training around how objective cognitive testing can enhance early detection of NCDs [2, 15, 37] above and beyond the clinical interview. Knowledge of the distinct domain of a patient’s cognitive deficits is required for the application of standard differential diagnostic criteria for many NCDs such as the Alzheimer’s syndromes, all primary progressive aphasia syndromes, and most other neurodegenerative disease syndromes [19, 20, 28–30, 38–41]. Many PCPs need support for recognizing how specific patterns of cognitive performance correspond to distinct NCD syndromes, which would improve their confidence in using cognitive tests for diagnostic purposes. Furthermore, programs to improve PCPs’ awareness of, and access to, the brief and streamlined cognitive assessment tools that are currently available may be needed. Bridging these important practice gaps through new training initiatives, guidelines, and policy changes around the diagnostic testing procedures will enable more PCPs to effectively administer and interpret cognitive tests themselves, substantially reducing costly and time-consuming referrals for specialized cognitive evaluation. These changes have the potential to ensure greater continuity of care for patients and their families.

Our study also showed underlying causes of practice difficulties in the use of brain imaging by PCPs in evaluating their patients with NCDs. The use of structural MRI to diagnose NCDs is a common standard in neurologic practice, and is a key element of differential diagnosis among different NCD syndromes [21, 22, 28, 29, 38, 40–46]. However, we found that PCPs were substantially less likely than specialists to order an MRI when evaluating a patient with an NCD (34% versus 69%). PCPs also reported low confidence levels in interpreting brain imaging findings (14% of PCPs reported high confidence compared to 70% of specialists), which may explain why PCPs are less likely to order MRIs. A discrepancy was also found between PCPs and specialists in the type of brain imaging ordered, which likely reveals important differences in their use of neuroimaging in NCD cases. During the NCD diagnostic process, an early step is to rule out non-neurodegenerative neurologic causes of neurocognitive symptoms, such as a brain tumor or stroke [19, 39]. This can be done fairly effectively with either CT or a more expensive structural MRI. However, MRI additionally allows for the identification of specific areas of focal degeneration [47], which is important not only to identify neurodegeneration, but also to differentiate among NCD syndromes. Brain CT scans are much less useful for this purpose [19, 28, 45]. Our study shows that PCPs ordered more CT scans than MRI scans for their NCD patients (41% CT versus 34% MRI). This data suggests that PCPs may be ordering imaging to rule out non-neurodegenerative conditions, but not to make a precise NCD diagnosis. To increase the confidence and capacity to provide specific and accurate diagnoses for their NCD patients, many PCPs may need clear guidelines and training as to which neuroimaging modality to prescribe given a patient’s particular presentation, as well as support for identifying the most typical patterns of regional atrophy that distinguish common NCD syndromes.

While it may be premature to expect many PCPs to become proficient at reading brain MRI scans, radiologists may also help PCPs if guidelines required them to provide more syndrome-specific diagnostic information in their clinical reports for patients with suspected NCDs. Automated volumetric analysis of MRI scans is routinely performed in clinical research settings, but translating these diagnostic analytics into clinical practice could dramatically improve PCPs ability to effectively utilize neuroimaging themselves. While there is some disagreement in primary care about which imaging tests are appropriate and which are extravagant [48], many specialists consider neuroimaging the foundation for the accurate differential diagnosis among NCD syndromes [19, 28, 47, 49]. Neuroimaging results, in turn, shape the course of subsequent patient care, including treatment decisions, prognosis, predicting disease course, and care planning.

Finally, our survey identified a general lack of familiarity among all providers with published diagnostic criteria for the various NCD syndromes. One-quarter of *all* providers stated that this lack of knowledge is a highly significant barrier to their evaluation and management of patients with NCDs. Clearly, training on existing diagnostic guidelines is needed to improve all providers’ knowledge and confidence around patient diagnosis. The underlying causes for this lack of comfort and knowledge may be remedied by routine exposure to standard diagnostic rules and decision support tools. If providers are more familiar with diagnostic criteria, they will in turn be more confident and effective in utilizing cognitive testing, imaging, and other key elements in their evaluations. This will lead to high quality prognostication and ongoing care for their patients with NCDs.

As of 2017, newly created Medicare billing codes in the United States have been established to allow providers more time with patients, and to provide better reimbursement when providers engage in comprehensive neurocognitive evaluation and management practices [50, 51]. Although this is a step toward remediation of systems-level practice barriers to improve quality of care, our results suggest that both PCPs and neurology specialists need more training and support in neurocognitive assessment methods and the choice of neuroimaging modalities in the evaluation of NCD patients.

### Limitations

The generalizability of this study is limited by the small sample size and bias due to participants self-selecting to respond to the survey. Additionally, PCP respondents work in many different practice environments (e.g. higher percentage of patients over 55, social worker/nurse practitioner/care manager on premises, socioeconomic status of population served), which may have differentially impacted their approach to dementia assessment and care. The small sample size limited our ability to determine the impact of these potentially confounding factors. Another important limitation is that self-perceived confidence is an imperfect proxy for actual effectiveness. For example, providers may indicate confidence in an area despite never actually implementing that practice, or they may be over-confident in their abilities but be very ineffective in practice. There may also be sampling bias because we offered payment for the completion of our survey. However, payment was minimal, and was accepted by our IRB as an amount that would not be considered coercive. Additionally, we did not pilot the survey prior to dissemination. Finally, while the practice characteristics of primary care providers surveyed in our study were mostly comparable to other large studies, our study had more providers reporting working in private practice [52].

## Conclusions

By 2050 more than 131 million people around the world will have dementia, and the prevalence of undetected dementia is high across the globe [53]. The current global costs of dementia are US $818 billion, which is expected to grow [54]. While our study focused on providers in the United States, to our knowledge there are no studies in global settings about provider confidence, attitudes, and practices in regards to specific aspects of the dementia evaluation performed. More work is thus needed to identify whether the issues described here are seen globally. This study illuminates a number of underlying causes for PCP’s lack of comfort and knowledge in the diagnosis and management of their patients with NCDs in the United States setting. These practice gaps were identified in several areas including cognitive testing, neuroimaging, and the knowledge of published diagnostic criteria for distinct NCD syndromes. Educational and practice-based interventions can remedy these gaps quickly. There is hope that such changes can realistically be achieved, given that PCPs are generally open to improvement and are eager to gain additional support guiding the decision processes around the identification, diagnosis, and care of their patients with NCDs.

## Supplementary information

**Additional file 1.** Provider Survey.

## Data Availability

The datasets used and/or analyzed during the current study are available from the corresponding author on reasonable request.

## Abbreviations

CT: Computed Tomography
MMSE: Mini-mental State Exam
MOCA: Montreal Cognitive Assessment
MRI: Magnetic Resonance Imaging
NCD: Neurocognitive disorder
PCP: Primary Care Provider
PET: Positron Emission Tomography

## References

[1] Alzheimer, Association, sciencestaff, alzorg. 2017 Alzheimer’s disease facts and figures 2017. doi:10.1016/j.jalz.2017.02.001.

[2] Bradford A, Kunik ME, Schulz P, Williams SP, Singh H (2009). Missed and delayed diagnosis of dementia in primary care: prevalence and contributing factors. Alzheimer Dis Assoc Disord.

[3] Shinagawa S, Catindig JA, Block NR, Miller BL, Rankin KP (2016). When a Little Knowledge Can Be Dangerous: False-Positive Diagnosis of Behavioral Variant Frontotemporal Dementia among Community Clinicians. Dement Geriatr Cogn Disord.

[4] Boustani M, Callahan CM, Unverzagt FW (2005). Implementing a screening and diagnosis program for dementia in primary care. J Gen Intern Med.

[5] Boise L, Camicioli R, Morgan DL, Rose JH, Congleton L (1999). Diagnosing dementia: perspectives of primary care physicians. Gerontologist.

[6] Boise L, Neal MB, Kaye J (2004). Dementia assessment in primary care: results from a study in three managed care systems. J Gerontol A Biol Sci Med Sci.

[7] Bradford A, Kunik ME, Schulz P, Williams SP, Singh H (2009). Missed and delayed diagnosis of dementia in primary care. Alzheimer Dis Assoc Disord.

[8] Brayne C, Fox C (2007). Dementia screening in primary care. Jama.

[9] Amjad H, Roth DL, Sheehan OC, Lyketsos CG, Wolff JL, Samus QM (2018). Underdiagnosis of dementia: an observational study of patterns in diagnosis and awareness in US older adults. J Gen Intern Med.

[10] Possin KL, Moskowitz T, Erlhoff SJ (2018). The brain health assessment for detecting and diagnosing neurocognitive disorders. J Am Geriatr Soc.

[11] Ngandu T, Lehtisalo J, Solomon A (2015). A 2 year multidomain intervention of diet, exercise, cognitive training, and vascular risk monitoring versus control to prevent cognitive decline in at-risk elderly people (FINGER): a randomised controlled trial. Lancet.

[12] Yaffe K (2018). Modifiable risk factors and prevention of dementia. JAMA Intern Med.

[13] 2019 ALZHEIMER’S DISEASE FACTS AND FIGURES Includes a Special Report on Alzheimer’s Detection in the Primary Care Setting: Connecting Patients and Physicians. https://www.alz.org/media/Documents/alzheimers-facts-and-figures-2019-r.pdf. Accessed May 20, 2019.

[14] Yokomizo JE, Simon SS, de Campos Bottino CM (2014). Cognitive screening for dementia in primary care: a systematic review. Int Psychogeriatrics.

[15] Hinton L, Franz CE, Reddy G, Flores Y, Kravitz RL, Barker JC (2007). Practice constraints, behavioral problems, and dementia care: primary care physicians’ perspectives. J Gen Intern Med.

[16] McCarten JR, Anderson P, Kuskowski MA, McPherson SE, Borson S (2011). Screening for cognitive impairment in an elderly veteran population: acceptability and results using different versions of the mini-cog. J Am Geriatr Soc.

[17] Boustani M, Perkins AJ, Fox C (2006). Who refuses the diagnostic assessment for dementia in primary care?. Int J Geriatr Psychiatry.

[18] Borson S, Chodosh J, Cordell C (2017). Innovation in care for individuals with cognitive impairment: can reimbursement policy spread best practices?. Alzheimers Dement.

[19] McKhann GM, Knopman DS, Chertkow H (2011). The diagnosis of dementia due to Alzheimer’s disease: recommendations from the National Institute on Aging-Alzheimer’s association workgroups on diagnostic guidelines for Alzheimer’s disease. Alzheimers Dement.

[20] Galvin JE, Sadowsky CH (2012). NINCDS-ADRDA. Practical guidelines for the recognition and diagnosis of dementia. J Am Board Fam Med.

[21] McCarthy J, Collins DL, Ducharme S (2018). Morphometric MRI as a diagnostic biomarker of frontotemporal dementia: a systematic review to determine clinical applicability. NeuroImage Clin.

[22] Filippi M, Agosta F, Barkhof F (2012). EFNS task force: the use of neuroimaging in the diagnosis of dementia. Eur J Neurol.

[23] Michie S, Johnston M, Abraham C, Lawton R, Parker D, Walker A (2005). Making psychological theory useful for implementing evidence based practice: a consensus approach. Qual Saf Heal Care.

[24] Mangurian C, Niu GC, Schillinger D, Newcomer JW, Dilley J, Handley MA. Utilization of the Behavior Change Wheel framework to develop a model to improve cardiometabolic screening for people with severe mental illness. Implement Sci. 2017;12(1). 10.1186/s13012-017-0663-z. 10.1186/s13012-017-0663-z PMC5686815 29137666

[25] Lyketsos CG, Lopez O, Jones B, Fitzpatrick AL, Breitner J, DeKosky S (2002). Prevalence of neuropsychiatric symptoms in dementia and mild cognitive impairment. JAMA.

[26] Woolley Khan BK, Murthy NK, Miller BL, Rankin KP (2011). JD. The diagnostic challenge of psychiatric symptoms in neurodegenerative disease. J Clin Psychiatry.

[27] Borson S, Frank L, Bayley PJ (2013). Improving dementia care: the role of screening and detection of cognitive impairment. Alzheimers Dement.

[28] Small G, Barry P, Buckholtz N (1997). Diagnosis and treatment of alzheimer disease and related disorders: consensus statement of the american association for geriatric psychiatry, the alzheimer’s association, and the american geriatrics society. JAMA.

[29] Rascovsky K, Hodges JR, Knopman D (2011). Sensitivity of revised diagnostic criteria for the behavioural variant of frontotemporal dementia. Brain.

[30] Strong MJ, Grace GM, Freedman M (2009). Consensus criteria for the diagnosis of frontotemporal cognitive and behavioural syndromes in amyotrophic lateral sclerosis. Amyotroph Lateral Scler.

[31] Folstein MF, Robins LN, Helzer JE (1983). The mini-mental state examination. Arch Gen Psychiatry.

[32] Moskowitz T, Rabinowitz N, Johnson E, et al. Neurology*.* Vol 86. Advanstar Communications; 2016. http://www.neurology.org/content/86/16_Supplement/P5.197. .

[33] Mielke MM, Weigand SD, Wiste HJ (2014). Independent comparison of CogState computerized testing and a standard cognitive battery with neuroimaging. Alzheimers Dement.

[34] Mielke MM, Machulda MM, Hagen CE (2015). Performance of the CogState computerized battery in the Mayo Clinic study on aging. Alzheimers Dement.

[35] Tsuruoka Y, Takahashi M, Suzuki M, Sato K, Shirayama Y (2016). Utility of the neurobehavioral cognitive status examination (COGNISTAT) in differentiating between depressive states in late-life depression and late-onset Alzheimer’s disease: a preliminary study. Ann General Psychiatry.

[36] Macaulay C, Battista M, Lebby PC, Mueller J (2003). Geriatric performance on the neurobehavioral cognitive status examination (Cognistat): what is normal?. Arch Clin Neuropsychol.

[37] Lathren CR, Sloane PD, Hoyle JD, Zimmerman S, Kaufer DI (2013). Improving dementia diagnosis and management in primary care: a cohort study of the impact of a training and support program on physician competency, practice patterns, and community linkages. BMC Geriatr.

[38] Gorno-Tempini ML, Hillis AE, Weintraub S (2011). Classification of primary progressive aphasia and its variants. Neurology.

[39] Geschwind MD, Shu H, Haman A, Sejvar JJ, Miller BL (2008). Rapidly progressive dementia. Ann Neurol.

[40] Chui HC, Victoroff JI, Margolin D, Jagust W, Shankle R, Katzman R (1992). Criteria for the diagnosis of ischemic vascular dementia proposed by the state of California Alzheimer’s disease diagnostic and treatment centers. Neurology.

[41] McKeith IG, Dickson DW, Lowe J, et al. Diagnosis and management of dementia with Lewy bodies: third report of the DLB consortium. Neurology 2005;65(12):1863–1872. doi:01.wnl.0000187889.17253.b1 [pii]. 10.1212/01.wnl.0000187889.17253.b1 16237129

[42] Armstrong MJ, Litvan I, Lang AE (2013). Criteria for the diagnosis of corticobasal degeneration. Neurology.

[43] Litvan I, Agid Y, Calne D (1996). Clinical research criteria for the diagnosis of progressive supranuclear palsy (Steele-Richardson-Olszewski syndrome): report of the NINDS-SPSP international workshop. Neurology.

[44] Albert Marilyn S., DeKosky Steven T., Dickson Dennis, Dubois Bruno, Feldman Howard H., Fox Nick C., Gamst Anthony, Holtzman David M., Jagust William J., Petersen Ronald C., Snyder Peter J., Carrillo Maria C., Thies Bill, Phelps Creighton H. (2011). The diagnosis of mild cognitive impairment due to Alzheimer’s disease: Recommendations from the National Institute on Aging-Alzheimer’s Association workgroups on diagnostic guidelines for Alzheimer's disease. Alzheimer's & Dementia.

[45] McKeith IG, Galasko D, Kosaka K (1996). Consensus guidelines for the clinical and pathologic diagnosis of dementia with Lewy bodies (DLB): report of the consortium on DLB international workshop. Neurology.

[46] Langa KM, Levine DA (2014). The diagnosis and management of mild cognitive impairment: a clinical review. Jama..

[47] Risacher SL, Saykin AJ (2013). Neuroimaging and other biomarkers for Alzheimer’s disease: the changing landscape of early detection. Annu Rev Clin Psychol.

[48] Things Physicians and Providers Should Question. Choosing Wisely. ABIM Foundation.

[49] Devous M (2002). Functional brain imaging in the dementias: role in early detection, differential diagnosis, and longitudinal studies. Eur J Nucl Med Mol Imaging.

[50] Borson S, Frank L, Bayley PJ (2013). Improving dementia care: the role of screening and detection of cognitive impairment. Alzheimers Dement.

[51] Graham J (2017). New toolkits help physicians detect, diagnose, and manage dementia. JAMA..

[52] Levine DM, Linder JA, Landon BE (2018). Characteristics and disparities among primary care practices in the United States. J Gen Intern Med.

[53] Lang Linda, Clifford Angela, Wei Li, Zhang Dongmei, Leung Daryl, Augustine Glenda, Danat Isaac M, Zhou Weiju, Copeland John R, Anstey Kaarin J, Chen Ruoling (2017). Prevalence and determinants of undetected dementia in the community: a systematic literature review and a meta-analysis. BMJ Open.

[54] Shah H, Albanese E, Duggan C (2016). Research priorities to reduce the global burden of dementia by 2025. Lancet Neurol.

