# Supplementary material for: Dementia assessment and management in primary care settings: a survey of current provider practices in the United States
Source: BMC Health Serv Res. 2019 Nov 29;19:919. doi: 10.1186/s12913-019-4603-2 (PMC6884754; doi:10.1186/s12913-019-4603-2)
Supplement: Supplementary file 1 — Additional file 1. Provider Survey. [file 12913_2019_4603_MOESM1_ESM.docx]

**Additional file 1.**

**UCSF/Quest Dementia Care Pathway**

**Outcomes Survey**

***Summary of proposed activities:***

Practitioners will be administered a standard online questionnaire that will help provide a baseline understanding of a number of quantitative and qualitative variables related to their clinical behaviors and attitudes towards evaluating and providing care for dementia patients. Special attention will be given to variables for which we anticipate the UCSF/Quest Dementia Care Pathway will be able to create measurable change.

- Neurocognitive disorder: a disorder of cognition or behavior that results from neurologic dysfunction due to injury or disease.
- Dementia: a neurocognitive disorder, often of neurodegenerative origin, that is severe enough to interfere with the ability to function at work and or other daily activities.
- Mild cognitive impairment: the early phase of a neurocognitive disorder before functional deficits become pronounced.

| 1. What is your medical specialty? ie, general internal med, family medicine, geriatrics, neurology, other 2. How would you describe your practice setting? (If more than one, rank the following according to the distribution of your time): Academic, HMO, ACO, Federally qualified health care center (FQHCC) or Community health center (CHC), Private individual practice, private group practice, other (please specify) 3. Zip code of primary practice location |
| --- |
| 1. How many years have you been in practice, post-residency? |
| 1. Which of the following types of formal training, if any, have you received in the past specific to the care of neurocognitive syndromes? (Fellowship, CME credits, professional education through community groups, e.g. Alzheimer’s Association, Other (please specify) or Never received any formal training in this area) |
| 1. On average, how many patients do you treat per month? |
| 1. Of the patients you see per month, how many are over the age of 55 (must see more than 10 patients over 55 y/o a month) |
| 1. What percentage of your patients are insured by Medicaid? 2. On average, how many patients do you newly diagnose for each of the following neurocognitive syndromes, per month? (Mild Cognitive Impairment, Dementia) |
| 1. Approximately, how many patients do you currently manage with each of the following existing neurocognitive syndromes? (Mild Cognitive Impairment, Dementia) 2. Do you use an EMR system at your primary practice location? |

**Section 1: Confidence/Baseline Satisfaction in Abilities:**

1. **How confident are you in your ability to correctly identify neurocognitive disorders?**

| 1 | 2 | 3 | 4 | 5 | 6 | 7 |
| --- | --- | --- | --- | --- | --- | --- |

Not at all Somewhat Moderately Extremely

confident confident confident confident

1. **How confident are you in your ability to diagnose specific neurocognitive syndromes (eg: semantic variant primary progressive aphasia, progressive supranuclear palsy, behavioral variant frontotemporal dementia, etc.)?**

| 1 | 2 | 3 | 4 | 5 | 6 | 7 |
| --- | --- | --- | --- | --- | --- | --- |

Not at all Somewhat Moderately Extremely

confident confident confident confident

1. **How confident are you in doing each of the following for patients with Alzheimer’s disease?**

*Subset questions for confidence in the following areas:*

1. Managing the general medical care of patients

| 1 | 2 | 3 | 4 | 5 | 6 | 7 |
| --- | --- | --- | --- | --- | --- | --- |

Not at all Somewhat Moderately Extremely

confident confident confident confident

1. Managing medication

| 1 | 2 | 3 | 4 | 5 | 6 | 7 |
| --- | --- | --- | --- | --- | --- | --- |

Not at all Somewhat Moderately Extremely

confident confident confident confident

1. Educating caregivers and families about dementia management and care

| 1 | 2 | 3 | 4 | 5 | 6 | 7 |
| --- | --- | --- | --- | --- | --- | --- |

Not at all Somewhat Moderately Extremely

confident confident confident confident

1. Conducting a genetic risk assessment for patients and families/ Discussing the results of genetic testing with patients and families

| 1 | 2 | 3 | 4 | 5 | 6 | 7 |
| --- | --- | --- | --- | --- | --- | --- |

Not at all Somewhat Moderately Extremely

confident confident confident confident

1. Prognosticating and providing stage-appropriate care

| 1 | 2 | 3 | 4 | 5 | 6 | 7 |
| --- | --- | --- | --- | --- | --- | --- |

Not at all Somewhat Moderately Extremely

confident confident confident confident

1. **How confident are you in doing each of the following for patients with specific neurodegenerative syndromes OTHER than Alzheimer’s disease? (e.g., semantic variant primary progressive aphasia, progressive supranuclear palsy, behavioral variant frontotemporal dementia, etc.)?**

*Subset questions for confidence in the following areas:*

1. Managing the general medical care of patients

| 1 | 2 | 3 | 4 | 5 | 6 | 7 |
| --- | --- | --- | --- | --- | --- | --- |

Not at all Somewhat Moderately Extremely

confident confident confident confident

1. Managing medication

| 1 | 2 | 3 | 4 | 5 | 6 | 7 |
| --- | --- | --- | --- | --- | --- | --- |

Not at all Somewhat Moderately Extremely

confident confident confident confident

1. Educating caregivers and families about dementia management and care

| 1 | 2 | 3 | 4 | 5 | 6 | 7 |
| --- | --- | --- | --- | --- | --- | --- |

Not at all Somewhat Moderately Extremely

confident confident confident confident

1. Conducting a genetic risk assessment for patients and families/ Discussing the results of genetic testing with patients and families

| 1 | 2 | 3 | 4 | 5 | 6 | 7 |
| --- | --- | --- | --- | --- | --- | --- |

Not at all Somewhat Moderately Extremely

confident confident confident confident

1. Prognosticating and providing stage-appropriate care

| 1 | 2 | 3 | 4 | 5 | 6 | 7 |
| --- | --- | --- | --- | --- | --- | --- |

Not at all Somewhat Moderately Extremely

confident confident confident confident

1. **How confident do you feel in your ability to use the following activities to help you diagnose different types of neurocognitive disorders? (**Rate each)
2. Interpret brain imaging findings

| 1 | 2 | 3 | 4 | 5 | 6 | 7 |
| --- | --- | --- | --- | --- | --- | --- |

Not at all Somewhat Moderately Extremely

confident confident confident confident

1. Interpret cognitive testing results

| 1 | 2 | 3 | 4 | 5 | 6 | 7 |
| --- | --- | --- | --- | --- | --- | --- |

Not at all Somewhat Moderately Extremely

confident confident confident confident

1. Understand how the patient history and symptom profile predicts specific neurodegenerative syndromes

| 1 | 2 | 3 | 4 | 5 | 6 | 7 |
| --- | --- | --- | --- | --- | --- | --- |

Not at all Somewhat Moderately Extremely

confident confident confident confident

**Section 2: Perceived Barriers to Care:**

**1. In your practice, how significant are the following barriers with respect to the evaluation and management of non-AD dementia syndromes?** (Rank all below)

1. Unfamiliarity with diagnostic criteria for different forms of neurodegenerative syndromes

| 1 | 2 | 3 | 4 | 5 | 6 | 7 |
| --- | --- | --- | --- | --- | --- | --- |

Not at all Somewhat Moderately Extremely significant significant significant significant

1. Insufficient time with patient

| 1 | 2 | 3 | 4 | 5 | 6 | 7 |
| --- | --- | --- | --- | --- | --- | --- |

Not at all Somewhat Moderately Extremely significant significant significant significant

1. Difficulty distinguishing neurodegenerative causes of dementia from other types of cognitive impairment

| 1 | 2 | 3 | 4 | 5 | 6 | 7 |
| --- | --- | --- | --- | --- | --- | --- |

Not at all Somewhat Moderately Extremely significant significant significant significant

1. Discomfort delivering a specific diagnosis

| 1 | 2 | 3 | 4 | 5 | 6 | 7 |
| --- | --- | --- | --- | --- | --- | --- |

Not at all Somewhat Moderately Extremely significant significant significant significant

1. Difficulty selecting which diagnostic tests are appropriate

| 1 | 2 | 3 | 4 | 5 | 6 | 7 |
| --- | --- | --- | --- | --- | --- | --- |

Not at all Somewhat Moderately Extremely significant significant significant significant

1. Lack of clarity about appropriate treatment plan

| 1 | 2 | 3 | 4 | 5 | 6 | 7 |
| --- | --- | --- | --- | --- | --- | --- |

Not at all Somewhat Moderately Extremely significant significant significant significant

1. Low reimbursement given cost of evaluation and management of patients with neurocognitive disorders

| 1 | 2 | 3 | 4 | 5 | 6 | 7 |
| --- | --- | --- | --- | --- | --- | --- |

Not at all Somewhat Moderately Extremely significant significant significant significant

**Section 3: Behaviors**

**1.** **How frequently do you (or your staff) refer a patient with suspected neurocognitive disorder for neuropsychological testing with a specialist**

1. never
2. less than 25% of patients with cognitive concerns
3. 25-50% of patients with cognitive concerns
4. 50-75% of patients with cognitive concerns
5. greater than 75%

**2. How frequently do you (or your staff) evaluate by other clinical methods (history and examination) but without standardized tools**

A. never

B. less than 25% of patients with cognitive concerns

C. 25-50% of patients with cognitive concerns

D. 50-75% of patients with cognitive concerns

E. greater than 75%

1. **How frequently do you (or your staff) refer the patient to a neurologist or other specialist for a dementia workup**

A. never

B. less than 25% of patients with cognitive concerns

C. 25-50% of patients with cognitive concerns

D. 50-75% of patients with cognitive concerns

E. greater than 75%

**4. How frequently do you (or your staff) administer a standardized cognitive screening test to your patients with cognitive concerns?**

A. never

B. less than 25% of patients with cognitive concerns

C. 25-50% of patients with cognitive concerns

D. 50-75% of patients with cognitive concerns

E. greater than 75%

F. I don’t administer it, but I use other people’s data

**5. How frequently do you administer standardized depression screen to patients with cognitive concerns?**

1. never
2. less than 25% of patients with cognitive concerns
3. 25-50% of patients with cognitive concerns
4. 50-75% of patients with cognitive concerns
5. greater than 75%
6. I don’t administer it, but I use other people’s data

**6. If no prior lab work has been done, how often do you include lab panels for reversible causes of cognitive impairment (B12, TSH, etc.)**

1. never
2. less than 25% of patients with cognitive concerns
3. 25-50% of patients with cognitive concerns
4. 50-75% of patients with cognitive concerns
5. greater than 75%
6. I don’t include them, but I use other people’s data

**7. If no prior imaging has been done, what percent of the time do you order the following imaging studies during a workup for dementia? (get percentages for each A-C)**

1. Head CT
2. Structural MRI
3. FDG PET

**8. Please rank each of the following factors in order of how strongly they influence your likelihood to refer to a neurologist? 1=highest, 4=lowest**

1. Type of neurodegenerative syndrome suspected
2. Greater severity of impairment
3. Strong family history suggestive of genetic cause
4. Complexity of co-morbid medical factors

**Section 4: Attitudes/Beliefs/Motivations**

1. **How useful do you find standardized cognitive screens for identifying whether your patient has a neurocognitive disorder? (e.g., Mini-mental State Exam [MMSE] or the Montreal Cognitive Assessment [MOCA])** 0-10; 0= Not at all useful 10= Extremely useful

| 1 | 2 | 3 | 4 | 5 | 6 | 7 |
| --- | --- | --- | --- | --- | --- | --- |

Not at all Somewhat Moderately Extremely useful useful useful useful

**2. Who most often administers cognitive screening tests to your patients?**

1. You
2. RN or NP
3. PA or other medical staff member
4. Neuropsychologist
5. Other ___________________

**3. Which of the following apply to you? (Please check ALL that apply.)**

1. I feel cognitive impairment can be adequately assessed through observation and/or informal memory tests during the clinical interview
2. I don’t have access to neuropsychological testing or I don’t know how to refer patients for neuropsychological testing
3. Obtaining outside neuropsychological evaluation is too expensive for my patients
4. Obtaining outside neuropsychological evaluation is too time consuming
5. Neuropsychological evaluation does not typically change my diagnosis
6. None of the above
7. **How useful do you find brain scans to be in making a dementia diagnosis?**

| 1 | 2 | 3 | 4 | 5 | 6 | 7 |
| --- | --- | --- | --- | --- | --- | --- |

Not at all Somewhat Moderately Extremely useful useful useful useful

1. **Which of the following apply to you? (Please check ALL that apply.)**
2. I’m unfamiliar/uncomfortable with interpreting MRI results
3. Obtaining neuroimaging is too expensive
4. Obtaining neuroimaging is too time consuming
5. Neuroimaging does not typically change my diagnosis
6. Neuroimaging doesn’t seem valuable because many guidelines don’t recommend it
7. None of the above
8. **How useful do you feel it is to screen for depression (with a questionnaire) during a workup for a neurocognitive disorder?**

| 1 | 2 | 3 | 4 | 5 | 6 | 7 |
| --- | --- | --- | --- | --- | --- | --- |

Not at all Somewhat Moderately Extremely useful useful useful useful

1. **How helpful would you find decision support (e.g., computerized interactive tools) to guide your choice of assessment tests for neurocognitive disorder (e.g., labs, cognitive, imaging, genetic testing)?**

| 1 | 2 | 3 | 4 | 5 | 6 | 7 |
| --- | --- | --- | --- | --- | --- | --- |

Not at all Somewhat Moderately Extremely helpful helpful helpful helpful

1. **How helpful would you find decision support (e.g., computerized interactive tools) to guide your detailed diagnostic process for specific neurodegenerative syndromes (e.g., lpvPPA, PSP, CBD, etc.)?**

| 1 | 2 | 3 | 4 | 5 | 6 | 7 |
| --- | --- | --- | --- | --- | --- | --- |

Not at all Somewhat Moderately Extremely helpful helpful helpful helpful

1. **How helpful would you find decision support (e.g., computerized interactive tools) to guide your treatment and care for patients with neurocognitive disorders?**

| 1 | 2 | 3 | 4 | 5 | 6 | 7 |
| --- | --- | --- | --- | --- | --- | --- |

Not at all Somewhat Moderately Extremely helpful helpful helpful helpful

**10. If you were confident that you had both the tools and the knowledge required to assess, correctly diagnose, and provide excellent ongoing care for all common neurodegenerative syndromes, how likely would you be to treat more of these patients in your practice without referring?**

| 1 | 2 | 3 | 4 | 5 | 6 | 7 |
| --- | --- | --- | --- | --- | --- | --- |

Not at all Somewhat Moderately Extremely likely likely likely likely
